# Southern Surgical and Gynecological Association

**Published:** 1892-01

**Authors:** 


### Society Reports.

### SOUTHERN SURGICAL AND GYNECOLOGICAL ASSOCIATION.*

FOURTH ANNUAL MEETING, HELD IN RICHMOND, VA., NOVEMBER 10, 11 AND 12, 1891.

### FIRST DAY—MORNING SESSION.

The Association convened in the hall of the Young Men's Christian Association, and was called to order by the President, DR. L. S. MCMURTRY, of Louisville, Ky., at 10 A. M.

Prayer was offered by the Rev. D. M. Hoge.

The first paper read was by Dr. J. W. Long, of Randleman, N. C., entitled

ALBUMINURIA; ITS RELATION TO SURGICAL OPERATIONS.

He drew the following conclusions:

1. That either ether or chloroform rarely ever injures healthy kidneys.

2. That when renal disturbances from the use of an anæsthetic, the kidneys being healthy, do occur, they are due rather to prolonged narcosis, exposure of the patient, or perhaps to the combined influences of the operation and the anæsthetic.

3. That a mild degree of albuminuria or nephritis, especially if recent, is not a contraindication to the use of chloroform or ether.

4. That even in the presence of advanced and extensive renal changes, an anæsthetic may be employed, provided the patient or family are advised of the additional risk.

5. That of the two anæsthetics usually employed, it is yet a mooted question as to which is the safer so far as the kidneys are concerned, unless it be in obstetrical operations.

*Received too late, and crowded out of December issue.

6. That, while it is by no means the rule, profound functional disturbances and even organic lesions may be induced by an operation, apart from the influence of the anæsthetic.

7. That such renal changes are due to reflex sympathetic action or sepsis, or both.

8. That operations in certain regions, notably the abdominal, genito-urinary, about the mouth and rectum, are especially liable to produce renal complications.

9. That a healthy condition of the kidneys, minimizes, but does not obviate, the dangers referred to.

10. That albuminuria is always indicative of renal lesions, and should be regarded with distrust; but is not a positive contra-indication to an operation.

11. That when albuminuria is associated with other evidences of advanced renal changes, no operation should be undertaken without first candidly stating to the patient, or friends, the dangers incident to the condition of the kidneys.

12. That paradoxical as it may seem, an operation will sometimes relieve an albuminuria due to acute affections.

13. That no surgeon is justified in undertaking an operation without first knowing the state of his patient's kidneys.

### SYSTEMIC INFECTION FROM GONORRHŒA.*

DR. BEDFORD BROWN, of Alexandria, Va., read a paper on this subject.

DR. J. EDWIN MICHAEL, of Baltimore, Md., read a paper entitled

### A REPORT OF SOME ADDITIONAL CASES OF EXTERNAL PERINEAL URETHROTOMY WITHOUT A GUIDE, in which he said that the operation is one of great value, both in gonorrhœal and traumatic cases, and he thinks one is justified in bringing forward any experience in it which may be of use to the profession. His results were very satisfactory, a fact which he attributes rather to the fortunate circumstances that his patients were largely free from grave constitutional disease than to any

*See page 607, December issue.

method or application which he had to suggest. He had simply followed what he considered the precepts of good surgery as applied to this region of the body, viz., free incision, free drainage, and as much of antiseptic surgery as the circumstances would allow.

The report of these eight cases brings up the number of patients on whom he made the operation of external perineal urethrotomy to seventeen, and the conditions which made the operation necessary include nearly all those which ordinarily indicate it. None of the patients have died at a period nearer than six months to the time of operation, and the deaths which have occurred were due to other causes. In the spring of 1887 he reported nine cases of perineal section without a guide, and he had only to add to the remarks made at that time, that increasing experience leads him to have more and more confidence in the good results of opening the perineum and less fear of danger.

It is true that he had had unusual good fortune in operating on cases which as a rule presented no grave kidney lesion, but while it must be admitted that such complication adds to the risk of the operation as much if not more than it does to others of equal gravity, he is firmly convinced that opening the perineum in old stricture cases with bad kidneys is much freer from danger than internal urethrotomy or even dilatation. A case in point: About ten years ago he did an internal urethrotomy on a patient with an old, tough stricture. In forty-eight hours he had a temperature of 107-8, and was very ill. The same patient returned to him a short time since. He could pass a No. 10 E. sound with difficulty. The stricture was resilient and closed after the sound to such an extent that urination was difficult and unsatisfactory. The patient had been having chills, and was somewhat nauseated and weak. His urine, although ammoniacal and ropy contained no evidence of grave kidney trouble. He proposed a combined internal and external urethrotomy, and refused to do either operation without the other. The patient consented. He opened the perineum freely and cut the urethra with the Otis instrument to 40 E. The temperature did not rise above 100°, the patient did well in every particular, and in three weeks he sent him home, passing a No. 36 F. instrument on himself.

FIRST DAY—AFTERNOON SESSION.

DR. W. F. WESTMORELAND, of Atlanta, Ga., read a paper entitled.

REDUCTION OF DISLOCATIONS BY MANIPULATION.

DR. JOSEPH PRICE, of Philadelphia, Pa., followed with a paper on

COMPLICATIONS IN PELVIC SURGERY, AND HOW TO DEAL WITH THEM.

The author's reasons for choosing this subject were that the importance of recognizing the part that complications play in the work of the surgeon is not appreciated by the generality of medical men, by general surgeons, and least of all, by the tyro in surgery, and by those who are anxious to begin their surgical investigations and trial-trips by an entrance into the domain of abdominal or pelvic surgery. The complications in this special branch of surgery are primarily those of surgery in general, with many things superadded to render them formidable. It may be the intention of the surgeon to remove the appendages for a bleeding fibroid. In ordinary operations the removal of the uterine appendages is to the skilled abdominal or pelvic surgeon one of the simplest of undertakings. If, however, he attempts to accomplish their removal without holding in mind the complications that as a rule exist, or if he is a neophyte or an experimental dabbler, he will find too late in many cases, that he has attempted an operation that he cannot finish, or if he does complete it, he has also sacrificed his patient, or rendered her worse off than before. In other words, to accomplish a cure, he must abandon removal of the appendages and perform hysterectomy, which has but little in common with the operation originally proposed. If this idea is still further carried out, we shall find that complications do not confine themselves to one system of organs, but extend to all surrounding structures by reason of inflammatory adhesions. This is true of the bladder, ureters, intestine, omentum, stomach and liver. Adhesions are the bane of abdominal and pelvic surgery, and hence we see that the greatest mistakes and failures are made by those who, from a knowledge of abdominal surgery simply, have attempted to deal with pelvic inflammations. The abdominal surgeons who can be counted as really successful pelvic surgeons are therefore few. This is said with no intention of detracting from the importance of adominal surgery. The strictly abdominal organs must always enter largely into the domain of surgery.

With regard to irrigation, we must get out of our heads the idea that it is dangerous. Too often in the writer's experience has hot water brought about a speedy reaction in patients whose lives were almost despaired of. We are told that cases do not need flushing, that they do badly under it. Dr. Price believes that they do need flushing if they are desperate cases, and if they do badly they do so not on account of the flushing, but because of the operation that preceded it. Next we have a resort in packing. Guaze packing accurately applied to the bleeding or oozing surfaces, so that it can be removed without interfering with the otherwise complete operation, is of infinite value in hemorrhage. It can be suffered to remain indefinitely almost, broadly speaking, at least, up to sixty or seventy hours, if absolutely clean and fresh, either salicylated or iodoformized. The drainage tube controls hemorrhage. The drainage tube is currently spoken of as if it were an annex to pelvic surgery, easily dispensed with. The writer uses it almost without exception in adhesion. His results are better than those obtained without its use.

The plea of the paper was for absolutely exact, painstaking work, that shall leave nothing for regret, nothing to do over, nothing to explain, but shall stand out in the light of results as justifiable, scientific and perfect, when put beside methods that palliate without curing, and are no more a part of real surgery than is hypnotism refreshing sleep.

LAPAROTOMIES PERFORMED DURING THE PAST YEAR.*

This was the title of a paper read by Dr. Thomas Opie, of Baltimore, Md.

*See page 577, December issue.

DR. CORNELIUS KOLLOCK, of Cheraw, S. C., read a paper entitled

### OVARIAN CYSTS, WITH THE REPORT OF A CASE OF OVARIOTOMY IN A YOUNG GIRL.

He said the causes of ovarian cyst seemed to be still a question *sub judice* in the minds of those who are most progressive, and who have made the greatest advancement in the science of gynecology. Various theories have been put forth by those of larger experience, who are earnest seekers after truth, and who are patient investigators of all unnatural and morbid phenomena. But no satisfactory decision has been obtained from all the patient and searching investigations that have been made as to the cause of this singular, unaccountable and sometimes fatal neoplasm, characterized by histological diversity from the viscus of which it is a production. Some of the theories seemed, at a glance, to be plausible, but upon close study we find they will not bear inspection.

CASE.—Miss C. L. H., aged 11 years, 8 months and 19 days; general health perfect in every particular. Menstruation first appeared about two months before she was 11 years of age, and continued with perfect regularity, never excessive or scant, nor was it accompanied by the slightest pain. Her physique was fine in every way. Though less than 12 years of age, she weighed 135 pounds; was strong and active. Her breasts were as full and large as those of a woman at 35. She was very handsome, had a fine voice and sang beautifully. She was very intellectual, and stood at the head in all her classes in a large high school. I saw her for the first time on the 9th of January, 1891. The abdomen was greatly distended, but facies ovariana was not very pronounced. I was confident she had an ovarian cyst, and I rather suspected she had two. On the 16th of January I made a section about three inches below the umbilicus and removed a cyst from each side, the one on the left weighing twelve pounds and that on the right seven pounds. A more prompt and complete recovery the writer had never seen from the simplest operation. Union by first intention took place, and the sutures, silver wire, were removed at the end of the 7th day. In twelve days she was up and about her room, and on the 23d day after the operation returned to her home, a distance of 200 miles.

It is now ten months since double ovariotomy was done on this young girl, and there has not been the slightest discharge from her of any kind. At each menstrual period there is considerable commotion in the pelvic region, attended with some uneasiness in the head and back, but at each period these symptoms decreased, and the last two were accompanied by no pain whatever. The remarkable physical development in this case still continues. It is now ten months since the operation. She has regained six pounds in weight, weighs 141 pounds and looks better than before she underwent ovariotomy. This young girl came from the purest and healthiest stock of people in this region. Not an individual on either side was ever known to have any constitutional trouble of any kind. Her mother and family physician, both highly intelligent, say they never knew her to be the least indisposed in any way.

---

### SECOND DAY—MORNING SESSION, November 11.

The Association was called to order by the President at 10 o'clock.

Prayer was offered by the Rev. Dr. Newton.

Dr. Thomas J. Moore, of Richmond, delivered an eloquent Address of Welcome.

Dr. William Warren Potter, of Buffalo, New York, read a paper entitled

#### A MEDICO-LEGAL ASPECT TO PELVIC INFLAMMATION, in which he said that pelvic inflammations in women have been described, discussed and debated from almost every point of view imaginable, until our periodical medical literature is flooded with articles on the subject, and medical society transactions are teeming and bristling with papers pertaining thereto. So far, however, he had not observed that any one had undertaken to discuss these intra-pelvic conditions from a medico-legal standpoint. It was his purpose to present that aspect of the question, taking for his text a case that developed an interesting problem in that respect.

After giving a history of the case, Dr. Potter emphasized the following points:

1. The intimate anatomical relations between the pelvic organs and the larger joints of the lower extremities, especially the hip and knee-joints, render them liable to reflexes.

2. The importance of careful diagnosis at the outset lest grave errors and possible disastrous consequences may result from treatment.

3. The medico-legal bearing that errors of judgment in diagnosis and treatment may have in relation to the patient, as well as upon the reputation of the physician.

### THE MEDICO-LEGAL ASPECT OF INTESTINAL SURGERY.

DR. JOHN D. S. DAVIS, of Birmingham, Ala., read a paper on this subject. He said many physicians and surgeons who condemn all mechanical aids for intestinal repair know not how to use them; never saw them used; refuse to indorse a resection for gunshot or stab wounds; have been known to go in the witness-box for purposes of condemnation and disproval when they knew no more about intestinal surgery than a wild Indian about school teaching.

In this day of specialties in medicine, but few general surgeons have either the appreciation, opportunity or disposition to qualify themselves as expert operators in intestinal surgery, but many, to the discredit of the profession, voluntarily appear in the criminal courts of the country pretending to be such, wise and proficient. One of the greatest professional sins of the day is perverted knowledge or conceited ignorance. It is too often that physicians and surgeons weaken and invalidate their opinions to a greater or less degree by unscrupulous interest in behalf of those employing them, a fact cunningly turned to advantage for defendants in criminal prosecution, and for like reason may become dangerous to the operators they oppose and envy.

To be able to do a laparotomy for stab or gunshot wounds of the intestines, inflicted by one with murderous intent, and be able to evade civil and criminal liability, the operator must (1) be able to show evidence of ordinary surgical knowledge in the requirement of the special operation to be performed; (2) he must possess ordinary surgical ability for doing the special operation to be performed; (3) he must exercise ordinary prudence in performing the special operation to be done, as to time, place, antiseptics, asepsis, assistance, nurses and after-treatment; (4) he must perform the special operation in an ordinary skillful manner. Hence, to prevent confusion, it will be good, if possible, to determine what constitutes ordinary surgical knowledge, ability, prudence and skill. Upon these depend the whole medico-legal status of the intestinal surgeon, and upon which the expert should be required to depend also. According to the practices and rulings of courts in this country the word *ordinary*, in its surgical adjectiveness, means that the surgeon shall be capable of and exercise that surgical knowledge, ability, prudence and skill with which a fair proportion of the surgeons of his given locality are endowed, and not that of the highest lights of his profession.

DR. JOHN A. WYETH, of New York City, made some remarks on

### ETHER ANÆSTHESIA.

DR. HOWARD A. KELLY, of Baltimore, Md., followed with a paper entitled

### HAND DISINFECTION.

DR. I. S. STONE, of Washington, D. C., read a paper on the

### PEDICLE IN HYSTERECTOMY.

The principal methods were described and illustrated by colored drawings, showing the arrangement of the pedicle in the abdominal wound. The author claims a revival of interest in the operation, and that there is need for its frequent performance. The statistics are far better now than ovariotomy claimed after it had become an operation of election, and was firmly planted in public favor.

Particular attention is given by the author to tying of the broad ligaments and the use of the elastic ligature. Sewing the parietal peritoneum to that of the pedicle in the extra-peritoneal cases was also dwelt upon.

The method by ventro-fixation had given good results in the author's hands, and served to accomplish two important purposes, viz., a speedy convalescence, and avoids the disagreeable sloughing which follows the use of the wire clamp. It may also be used in some cases of short pedicle, where the wire may not easily be applied. The methods were compared and statistics furnished, showing that the extra-peritoneal method with wire and pin gave better results than either of the others. That ventro-fixation came next, and the intra-peritoneal method last, with a large mortality. A method of closing the capsule over the stump was described, which the author claimed would answer for either *dropping* it, or sewing in the wound—ventro-fixation. In the latter case the suspensory sutures are placed and the pedicle sewed in and under the lower end of the abdominal incision. Great care is required in closing the capsule over the raw surface of the stump so that separation may not occur. Owing to the peculiar contractile nature of the capsule care must be taken to leave sufficient length for approximation of peritoneal surface.

The uterine arteries are to be tied in any case when hemorrhage is likely to occur, and drainage may be required. Besides reference to methods the author described the process through which the womb passes subsequent to supra-vaginal hysterectomy.

All myomatous tissue should be removed, which can only be effected in some cases by a process of reduction of the pedicle. This is very important, as in the operation where a large amount of myoma is left, more time is required for atrophy and absorption to reduce the pedicle to its proper size. Great danger to the patient is apt to follow, where a broad base of the tumor is left in either method of treatment, because this mass must be disposed of before the patient entirely recovers.

The author had observed a sufficient number of cases to declare that permanent fixation of the stump to the abdominal wall was the rule, where the extra-abdominal methods were used, and especially when the broad ligaments were cut away to prevent traction.

---

SECOND DAY—AFTERNOON SESSION.

The Association was called to order by the First Vice-President, DR. J. MCFADDEN GASTON, of Atlanta, Georgia.

The first thing in order was the Annual Address of the President, by DR. L. S. MCMURTRY, of Louisville, Ky. Dr. McMurtry selected for his subject

A PLEA FOR PROGRESSIVE SURGERY.

He said within fifteen years the entire practice of surgery has been revolutionized. New methods have been introduced, and new regions invaded. Comparatively recent teachings have become obsolete in practice, and modern treatises recast. The science and art of gynecology, which a few years since was limited to a small and narrow field, has grown into a great branch of medical science and practice. Formerly divided between midwifery and surgery, as a minor branch of one or both, gynecology has become an independent and essential department of the healing art.

When Marion Sims announced through the columns of the *British Medical Journal* that he believed the proper course of treatment in every case of gunshot wound of the abdomen is to open the stomach, search for the bleeding points and secure them, and suture intestinal perforations, he was pronounced by many eminent surgeons to be a dreamer. The suggestion of Sims was most timely, and shortly afterwards Bull successfully executed the operation. For years the treatment of opium in full doses had been pursued, with death in waiting. Now there is scarcely a State in the Union that one or more patients have not been rescued from certain death by prompt resort to operative treatment. He mentioned these circumstances to illustrate and emphasize the point, that surgery is advanced more by the aggressiveness of the surgeon than by timidity. In the face of desperate conditions of disease and injury, where there can be no safety whatever in delay and palliation, the only treatment worthy of consideration is the aggressive course which promises success. Under such conditions the most heroic surgery is conservative, and any other course is not conservative.

DR. THOMAS ADDIS EMMET, of New York, read a paper entitled

INJURIES TO THE PELVIC FLOOR AND THE METHOD OF REPAIRING THE SAME.

DR. JOSEPH TABER JOHNSON, of Washington, D. C., followed with a paper on

THE GROWTH OF FIBROID TUMORS OF THE UTERUS AFTER THE MENOPAUSE.

He said the object of the paper was to put on record cases and opinions in opposition to this view of this important subject, and to aid in recasting our views and in modifying our practice.

He had within the past five years seen at least a dozen women with large growing and troublesome fibroid tumors of the uterus, who were over fifty years of age; some of them over sixty. These women had been assured by their physicians that if they could get along somehow until after the change of life their tumors would not only stop growing, but that they would lessen in size, and probably go away altogether; at least the troublesome and dangerous symptoms would disappear. They had been advised against any radical operation, and encouraged to believe that as they grew older they would get entirely well. In perhaps the majority of cases this might prove to be very good advice; but the point which the author wishes to make is, that as we are now better acquainted with the history and behavior of these tumors, that this is no longer safe advice to give. We cannot assure any woman that her tumor may not prove to be one of the exceptional cases, and that it may not grow more rapidly after the menopause than it did before, or that it may not present complications equally distressing and disastrous. When from forty to fifty per cent. of women subjected to supra-vaginal hysterectomy died from the effects of the operation, this was very safe and conservative counsel to follow. The possible dangers of the tumor were not equal to the probable dangers of the operation.

The author drew the following conclusions:

1. That the "rule" stated in the text-books, that uterine fibromata cease to grow after the menopause, has many more exceptions than is generally supposed.

2. That *when* they continue to grow after the menopause, they pursue a more disastrous course than before.

3. They more frequently become cystic, calcareous or have abscesses developed in them.

4. These conditions requiring operation according to well-known rules of surgery, the patients are in a less favorable condition for recovery than before the monopause.

5. If the above conclusions are admitted to be true, it must follow that they furnish additional indications for more frequent and earlier resort to the radical operation.

In the hands of the best operators in cases where a pedicle can be secured, the mortality of supra-vaginal hysterectomy is rapidly approaching that of ovariotomy.

### THE SURGICAL TREATMENT OF ANTERIOR DISPLACEMENTS OF THE UTERUS.

DR. C. A. L. REED, of Cincinnati, Ohio, read a paper on this subject. He said, anterior displacements of the uterus, when they exist to the pathological degree, are the opprobria of the gynecic art. It is indeed true that many wombs lean far forward without inducing symptoms, but it is likewise true that many of them that are thus malposed do entail symptoms, objective and subjective, that frequently baffle our resources. It is a misfortune, too, that in many of all the displacements to which the womb is liable, those in which the organ deviates anteriorly to the normal axis are vastly the more prevalent. Thus in an aggregate of four hundred and ninety-four cases by Nonat, Meadows, Scanzoni, Valleix and Hewitt, quoted by Thomas and Munde, there were two hundred and ninety-four ante-flexions, and one hundred and eighty retro-flexions; while Munde himself reports two hundred and ninety-four ante-flexions, thirty-three retro-flexions and ten latero-flexions in a total of three hundred and thirty-seven cases. As the latter authority is disposed to look upon ante-flexions in their minor stages as a physiological (even congenital) condition, it is legitimate to infer that his statistics are based upon observations of displacements in the pathological degree. The conclusion is forced upon us, then, that of all the displacements of the uterus, those of the anterior variety are the more frequent; while the records of practice will force us, likewise, to the conclusion that of all the womb displacements those of the anterior variety are less amenable to treatment than are any of the others.

In the treatment the term *surgical* is employed in contradistinction to any method of treatment by pessaries, tamponnade, or electricity. It may be premised that all surgical methods devised for the relief of these conditions should be directed, first, to the removal, when practicable, of the causes of the diseased conditions proper, and, finally, to the readjustment of the diseased organs to the normal physical forces of the pelvis.

In conclusion the author desired the Association to consider:

1. The etiological relationship of contracture of the utero-sacral ligaments to ante-flexion.

2. The possibility of overcoming this condition by such conservative measures as rest, pelvic depletion and appropriate manipulations.

3. The feasibility of removing the obstructive dysmenorrhœa and the sterility usually incident to these cases by the plastic operation which he had described.

4. The inexpediency of forcible dilatation for the relief of these cases and its inability to effect a permanent cure.

### THE PART THE SHOULDERS PLAY IN PRODUCING LACERATION OF THE PERINEUM, WITH SUGGESTIONS FOR ITS PREVENTION.

This was the title of a paper read by Dr. W. D. Haggard, of Nashville, Tennessee, in which he made the following suggestions:

1. The patient should occupy the left lateral decubitus, at least during the second stage of labor.

2. Overcome rigidity of the vulvar outlet by the judicious use of chloroform.

3. The presenting part of the child should be supported and not the perineum during the passage of the head and shoulders.

4. Support the head by pressing it well up under the symphysis pubis by placing the right thumb in the rectum and fingers of right hand expanded over the occiput.

5. To retard the exit of the shoulders, pressure should be applied to the trunk and shoulder by placing the index and middle fingers of the left hand in the rectum with the thumb in the vagina to restrain its exit.

6. Support the head and neck by pressure well over the symphysis pubis.

---

THIRD DAY—MORNING SESSION, NOVEMBER 12.

The Association was called to order by the President at 10 A. M.

DR. JAMES A. GOGGANS, of Alexander City, Ala., read a paper entitled

ABDOMINAL SECTION IN A CASE OF CYST OF THE MESENTERY.*

---

THINNESS OF UTERINE WALLS SIMULATING EXTRA-UTERINE PREGNANCY WITH REPORT OF TWO CASES, was the title of a paper by DR. GEO. J. ENGELMANN, of St. Louis, Missouri.

The author said there are many difficulties in the way of a positive diagnosis of early pregnancy, even in cases surrounded by conditions less unusual, but they assume alarming proportions when aggravated by the curious complications which may arise in individual cases, above all when conditions are simulated in which delay is dangerous and operative interference seems called for; when a decision is urgently demanded, a decision upon which a life, and perhaps two, may depend. Whilst the auditor may criticize at his leisure and readily differentiate the conditions depicted, it is only he who is to pronounce and to act who can realize the difficulties of this entangling and so knotty problem.

*To be published in this journal.

CASE I.—Patient, 32 years of age, had borne three children in the six and a half years of her married life, the youngest twenty months ago, which she was still nursing, and the menstrual flow has not as yet reappeared since the birth of this child. The patient came to his clinic for relief from a variety of discomforts from which she had been suffering more or less for the past three months. She complains of sick headache, vomiting spells, fulness of the stomach, belching after meals and an intermittent swelling of the abdomen ; a pain in the groin, appearing before such swelling and a small tumor above the right groin, which she had first noticed three weeks ago and as she stated, "then suddenly made its appearance." An examination revealed large varicose veins over the lower limbs; a solid, round, movable tumor above symphysis and right groin; the cervix low and large; the uterine body thickened, lying low in the pelvis, with a certain mobility independent of the superimposed tumor; an applicator entering three and a half inches slightly *ante*. Notwithstanding the wine color of the pronounced cystocele and the cervix, pregnancy seemed out of the question, and the tumor was diagnosed as most probably a dermoid of the right ovary, hardly one connected with the uterine wall. In the course of an examination two weeks later, a very different condition of affairs was revealed. The tumor had disappeared, and a fœtus was found in the utero-vesical space, freely movable, apparently floating about, the small parts being distinctly felt as if underneath a wet towel both through the vagina and abdominal walls. So distinct did the small parts appear to the examining finger, that it seemed impossible to realize that even as much as a thickness of the vaginal tissues should intervene, and the abdominal walls must certainly have been very much attenuated to disclose the fœtal parts with such distinctness. Probe showed the uterine cavity free six and a half inches in length, still slightly *ante*, but never curving forward in the direction of the previous tumor.

The treatment for the supposed subinvolution was discontinued, the patient warned to keep quiet and to notify Dr. Engelmann upon the occurrence of any abnormal symptoms. He believed the case to be one of ectopic gestation, either within the broad ligamentor in the abdominal cavity after tubal rupture marked by the sudden appearance of the tumor five weeks ago, yet he was not sufficiently positive to warrant the immediate resort to the knife, and well that he did not do so, as persistent treatment and repeated examinations resulted in labor pains and the delivery of a five months' fœtus in the most correct and natural manner.

Dr. Robert T. Morris, of New York, contributed a paper on

THE REMOVAL OF NECROTIC AND CARIOUS BONE WITH HYDROCHLORIC ACID AND PEPSIN.

The author said sometimes it is desirable to remove dead bone without subjecting a weak patient to a dangerous or deforming operation. Attempts have been made with some success at clearing out this bone by a process of decalcification, but there are two chief reasons why failures have resulted as a rule. In the first place, it was discovered that superficial layers of dead bone were decalcified easily enough, but the acids did not reach deeply through the mass, especially if portions were infiltrated with caseous or fatty débris. In the second place, cellulitis was pretty apt to develop during the course of treatment.

After much experimentation he had finally adopted the method of work which seemed to be complete. An opening is made through soft parts by the most direct route to the seat of dead bone, and if sinuses are present they are all led into the one large sinus, if possible. The large direct sinus is kept open with antiseptic gauze, and the wound allowed to remain quiet until granulations have formed. Granulation tissue contains no lymphatics and absorption of septic material through it is so slow that we have very good protection against cellulitis. The next step consists in injecting into the sinus a two or three per cent. solution of hydrochloric acid in distilled water. If the patient is confined to bed, the injections can be made at intervals of two hours during the day; but if it is best to keep the patient up and about, the acid solution is thrown into the sinus only at bed-time. In either case the patient is to assume a position favorable for the retention of the fluid. Decalcification of exposed layers of dead bone takes place quickly, and then comes the necessity for another and very important step in the process. At intervals of about two days an acidulated pepsin solution is thrown into the sinus (he uses distilled water, ℥iv; hydrochloric acid, m. xlv; Fairchild's pepsin, ʒss), and this will digest out decalcified bone and caseous and fatty debris in about two hours, leaving clean dead bone exposed for a repetition of the procedure. The treatment is continued until the sinus closes from the bottom, showing that the dead bone is all out.

Even in distinctly tuberculous cases the sinuses will close if apparatus for immobilizing diseased parts and tonic constitutional treatment are employed, as they should be, in conjunction with our efforts at removing the dead bone. If suppuration is free in any cavity in which we are at work, it is well to make a continual practice of washing out the cavity with peroxide of hydrogen before each injection.

It is a popular impression in the profession that living bone is not attacked by dilute mineral acids, but as it makes a good deal of difference whether the impression is correct or not, he experimented as follows: A portion of the keratinoid layer was removed from the carapace of a turtle (nanemys guttatus), and the animal was then placed tail downward in a glass of five per cent. hydrochloric acid solution. In the glass he placed also a segment snipped from the plastron of the turtle, and a transverse segment from an old dry humerus of a man. The piece of humerus was completely decalcified in six hours; the segment from the plastron was soft in about twenty hours and the carapace of living bone was decalcified at the exposed part in thirty hours. He was then curious to know what effect the acid had had upon the blood vessels of the decalcified bone, and Dr. Smith of the laboratory of the Post-Graduate Medical School made for him several sections of the carapace, which included both decalcified and healthy bone. Investigation showed that all of the blood vessels were destroyed wherever the bone was softened, and the action of the acid had extended farther up along the larger blood vessels than elsewhere.

The difference in time between decalcification of the dead bone, six hours, and of living bone, thirty hours, is significant; a five per cent. solution of the hydrochloric acid having been used. If we use a two or three per cent. solution of hydrochloric acid, a wall of lymph and of granulation tissue is thrown out upon the surface of the living bone for protection, and only dead bone is attacked. This at least has been his observation in several cases in which the results of treatment could be easily watched.

DR. LANDON CARTER GRAY, of New York, in a paper entitled

THE PRESENT STATUS OF CEREBRAL SURGERY, touched upon the modern aspect of intra-cranial surgery. The speaker first passed in review our present knowledge of localization of functions of the brain, stating that we are well acquainted with the functions of the motor area, of the third frontal convolution, the frontal lobe, the Island of Riel, the two upper temporal convolutions, the cuneus, certain portions of the basal ganglia, the base of the brain and the cerebellum, and that we knew nothing, or had still under discussion the question of the localization of the centers for the sensations of touch, pain, muscular sense, temperature sense, most of the parietal lobe, and most of the tempero-sphenoidal lobe with the exception of the olfactory lobe. He stated that operations for fracture of the skull with or without hemorrhage, for abscess, and for tumors that were removable and localizable were usually successful; those for so-called idiopathic epilepsy were utterly valueless, as were also those for epilepsy supposed to be due to genital or ovarian irritations, whilst those done for epilepsy due to removable and localizable lesions of the intra-cranial contents were usually successful so far as the lesion was concerned, although it was a grave question as to whether the epileptic habit was ever cured. The latest operation for idiocy supposed to be due to premature ossification of the fontanelles was still under discussion and consideration, the cases being too few and too recent to permit of any conclusion; whilst the operations for hydrocephalus and for epilepsy due to such early infantile and fœtal lesions as parencephalus, hemorrhage and meningitis were inde-

[illegible] H urther impressed upon surgeons the great difficulty that there often was in finding a sub-cortical lesion of the centrum ovale that was deep seated or small, and the fact should be borne in mind that there might be no decussation of the motor fibres from the hemispheres, so that a lesion would be found upon the same side as the paralysis.

### SOME COMPLICATIONS OF PSOAS ABSCESS.

This was the title of a paper read by Dr. J. McF. Gaston, of Atlanta, Georgia.

---

### THIRD DAY—AFTERNOON SESSION.

DR. PAUL B. BARRINGER, of Richmond, Va., read a paper on

### VENOMOUS SERPENTS OF THE UNITED STATES AND THE TREATMENT OF WOUNDS INFLICTED BY THEM.

DR. CHRISTOPHER TOMPKINS, of Richmond, Va., followed with a paper entitled

### A CASE OF INDUCED ABORTION FOR RELIEF OF NAUSEA AND VOMITING, WITH REMARKS.

On August 1, 1885, he was called to see Mrs. J., aged twenty-four, and as nearly as could be ascertained, three and a half months pregnant with her first child. Patient was born in the mountainous part of Virginia; she had led an active outdoor life and grew up to be a woman of good height and of round full figure. January 14, 1884, she married.

While in the city of New Orleans, in stepping from the platform of a car, she sprained her ankle. This, although treated immediately by a physician of that place and subsequently in this city, caused her great suffering. Finally, refusing to yield to the usual treatment, the part was put in a plaster cast; she went about on crutches, and after many months recovered. In the meantime she became pregnant and from the first was attacked with nausea and vomiting. Mild in the beginning, it gradually increased in gravity, till she sent for him on the 1st of August, 1885.

Her husband stated that she had had fever for two weeks. He found her in bed and learned that she had been there for days; her figure not robust, and her face thin and attenuated. What little she had eaten in the past ten days or two weeks had been apparently rejected, her temperature one degree above the normal; tongue foul; sordes on the teeth, and the breath of a sour and bilious odor. The pulse was fairly good, considering her condition. Even the mention of food was distressing to her, and the sound of the dinner bell, though far off from her, caused such distress that its ringing was discontinued by the family. The bowels had throughout her pregnancy been constipated, only moving once in two or three days. Although continually retching, very little or no blood had been seen in the material vomited, except on two occasions, and then not a great deal, and such as there was was of a florid, scarlet color. No medicine has been given and no treatment taken, except the occasional use of lime water, which she said "did no good."

The patient did not improve up to August the 7th, when Dr. Tompkins, thinking the case one of the greatest gravity, and that the question of abortion could no longer be deferred, invited Drs. J. B. McCaw and Aaron Jeffrey to meet him in the afternoon in consultation. All agreed that abortion must be produced, in order to give the patient a last chance for her life, which was done.

*Remarks.*—The case is reported principally because it was an unsuccessful one, and because he wished to disabuse the minds of those who are not experienced in such operations of the notion, commonly believed and often expressed, that the induction of abortion for the nausea and vomiting of pregnancy is in skillful hands an undertaking devoid of danger and necessarily attended by success. In this case he is of the opinion that death was the result of the protracted debility and an enfeebled constitution, due to her long confinement and suffering; first, from the injury to her ankle, from which she had not recovered when she became pregnant and was attacked by nausea and vomiting, this last continuing till her death. Under such circumstances the outlook was indeed very unfavorable, for to the shock of operation and depression incident to the use of chloroform, there was added fever and protracted prostration, both from injury to the ankle and from want of nutrition, the result of the long existing nausea and vomiting. He had before and since operated on women for the nausea and vomiting of pregnancy, and with success, whose apparent condition was much worse than that described in the above case, but without the history of a previous injury or disease.

The prognosis, always unfavorable, ought, when the case is so complicated, to be of the most guarded kind. The practitioner should not, however, hold his hands on this account, for the operation affords the poor sufferer the only opportunity of relief. The author uses metal dilators instead of tents, and completes the operation at one sitting. He is likewise convinced that the least possible chloroform used the better the result.

The following officers were elected:

*President*—Dr. J. McFadden Gaston, Atlanta, Ga.

*First Vice-President*—Dr. Cornelius Kollock, Cheraw, S. C.

*Second Vice-President*—Dr. Geo. Ben Johnston, Richmond, Va.

*Secretary*—Dr. W. E. B. Davis, Birmingham, Ala.

*Place of Next Meeting*—Louisville, Ky., second Tuesday in November, 1892.

*Chairman of Committee of Arrangements*—Dr. L. S. McMurtry, Louisville, Ky.

---

CARBUNCLES.—Dr. A. E. Spohn, writing in the *Medical and Surgical Reporter*, states that he has had excellent results in the treatment of carbuncle by the external use of a ten per cent. solution of chloral hydrate in glycerine and water, applied constantly by means of absorbent cotton, combined with the internal administration of sulphide of calcium. He was led to use the chloral externally to relieve the pain, and was surprised to find that it also had curative effect.—*Exchange.*

